# A Proposition of an In Situ Production of a Blended Cement

**DOI:** 10.3390/ma13102289

**Published:** 2020-05-15

**Authors:** Jacek Halbiniak, Jacek Katzer, Maciej Major, Izabela Major

**Affiliations:** 1 Faculty of Civil Engineering, Czestochowa University of Technology, Dabrowskiego 69, 42-201 Czestochowa, Poland; jacek.halbiniak@pcz.pl (J.H.); maciej.major@pcz.pl (M.M.); izabela.major@pcz.pl (I.M.); 2 Faculty of Geoengineering, University of Warmia and Mazury in Olsztyn, Heweliusza 4, 10-720 Olsztyn, Poland

**Keywords:** cement production, recycling, white ceramic, cement, waste

## Abstract

Many byproducts and waste materials with pozzolanic properties can substitute natural raw materials in cement production. Some of these waste materials like fly ash and blast furnace slag are commonly harnessed by cement industry. Others are of seldom use due to limitations of the very centralized cement production systems currently in use. In the authors opinion, it is necessary to change this system to enable efficient utilization of various waste materials that are available locally (e.g., white and red ceramics). In this study, a new partially centralized system of cement production is proposed. The adoption of a new system would significantly reduce the volume of long-distance transportation and enable utilization of numerous locally available waste materials that are currently dismissed. The last stage of production of the ready-to-use cement would take place in situ. The cement would be produced on demand and be immediately used for concrete production on-site. The research program was conducted considering the importance of the quality of cements obtained in the new way, substituting up to 12% of its mass by white ceramics. The research program was proof of concept of the proposed cement production system. It was shown that the quality of “in situ cement” does not differ from standard cements.

## 1. Introduction

The cement industry is one of the most energy-intensive sectors of the global economy and has an enormous impact on the volume of carbon dioxide emissions. It is estimated that cement production consumes around 15% of the total energy demanded by global industry [1,2]. Production of one ton of common Portland cement (CEM I) according to EN 197-1 releases up to 900 kg of CO_2_ [3,4]. The highest CO_2_ emission arises during the production of Portland clinker during calcination (decomposition of CaCO_3_). As a result of this process, calcium oxide (CaO) and free carbon dioxide are released. The calcination process occurs in a rotary kiln at temperature about +1450 °C. To achieve such high temperatures in the kiln, significant amounts of coal and (increasingly more often) alternative fuels are burnt, emitting CO_2_. Currently, worldwide cement production is considered to be responsible for approximately 7.4% of the global anthropogenic CO_2_ emissions (2.9 Gt in 2016) [5]. Altogether, cement production consumes huge amounts of various types of fossil fuels and huge volumes of natural minerals, rich in CaCO_3_ (e.g., limestone, clay, marble, chalk) and emits significant amounts of greenhouse gases. According to the US Geological Survey, in 2018 the production of cement by the top twelve countries reached 3.3 billion tons [6]. In the coming years, cement demand will increase. It will be associated with an increase of greenhouse gas emissions, consumption of natural minerals and natural fuels. All these factors will dramatically impact the environment. Therefore, investing effort in research to reduce the impact of cement production on environment is necessary more than ever.

Currently in the EU, the majority of used cements are blended cements consisting of—apart from clinker—multiple waste materials with pozzolanic properties. The most common waste materials used for blended cement production are fly ash, blast furnace slag, silica fume, lime powders, etc. Different research teams have successfully experimented with using multiple other locally available waste materials (such as: red and white ceramic [7,8,9,10], marble dust [11], marble sludge [12], rice husk [13,14,15], volcanic ash [16], pumice powder [16], sea shells from mussels [17], waste glass powder [18,19] and wastes from the production of mineral wool [20]) for blended cement production. All previous studies show the feasibility of utilization of locally available waste materials for blended cement production. However, such local trials and demonstrators are rarely adopted by large cement producers. The current system of cement production is not suited for harnessing varied locally available waste materials. In some cases, the reduction of energy consumption and emissions is questionable for the local waste material used in blended mixtures due to long-distance transportation. In authors opinion, a new philosophy of cement production is needed to enable widespread of harnessing numerous locally available waste materials for blended cement production. In other words, the cement industry should be reorganized from a solely centralized production system to a partially centralized production system. In this study, both systems of the cement industry are discussed. The proposed partially centralized production system would enable sustainable, easy and efficient utilization of numerous locally available waste materials for blended cement production. In regions where pure Portland cement has a dominant market share (due to numerous reasons) new possibilities of blended cement production would be enabled. The proposed concept is supported by a relatively small research program demonstrating key elements of the new blended cement production philosophy.

## 2. Reasoning behind the In Situ Production of a Blended Cement

**Figure 1:**
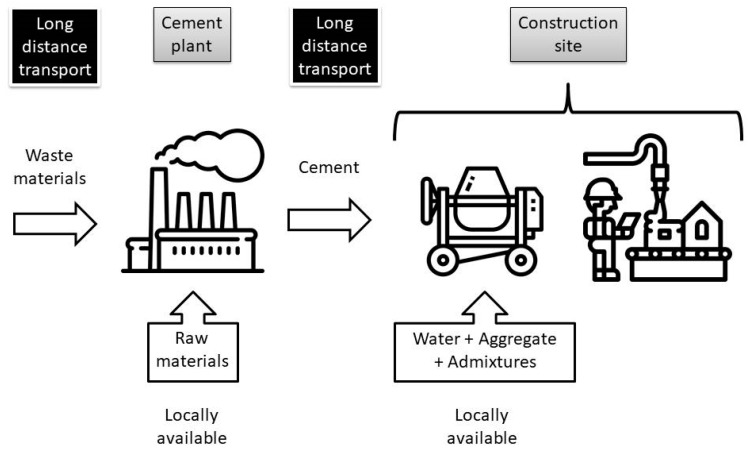
Current cycle of materials circulation during cement and concrete production (see Acknowledgments).

Cement plants are large industrial facilities usually located near deposits of natural raw materials used for the production. In this way, the long-distance transport of large volumes of materials needed for cement production is axed. The only part of the cement plant operations associated with long-distance transport is shipping of produced, packed and ready-to-use cement. This philosophy of production was valid and energy efficient at the end of 19th century and for the first half of the 20th century when only pure Portland cement was produced. Roughly 70 years ago the process of adding waste materials to clinker for cement production was started. In this way blended cements were introduced to the market. Initially, fly ash and blast furnace slag were used to partially substitute clinker. Over the years multiple other waste materials were adopted for blended cement production. Substituting clinker by waste materials was originally aimed to make the cement industry greener but some key aspects of the production and shipping process were forgotten in the process. Location of available waste materials is quite random and very often distant from a cement plant [21,22,23]. One has to transport them for long-distances to a plant. Keeping in mind that volume of waste materials in blended cement varies from 14% (CEM II) to over 50% (CEM V—up to 80%), long distant transporting of these materials play a key role in general sustainability of cement production. In Figure 1 a scheme of current materials circulation during cement production and its shipment is presented. Long-distance transportation of huge volumes of materials is needed twice in a current model. The first long-distance transportation is needed to deliver all waste materials used for blended cement production. These materials can constitute over 50% of produced cement (e.g., for production of CEM III, CEM IV and CEM V according to EN 197–1) thus environmental impact of their transportation is significant. The second long-distance transportation is associated with shipment of ready-to-use cement to a building site or concrete ready-mix manufacturer. This time waste materials constituting significant volume of blended cement are transported for long-distances for the second time. The current production system of cement, apart from being ineffective in terms of long-distance transport, obstructs utilization of numerous waste materials available locally.

The centralized system of cement plant operations enables only consumption of waste materials available in very large quantities located in specific places. Research effort focused on possible utilization of much wider and diverse range of locally available waste materials for blended cement production is not possible to commercialize in this system. Therefore, a radical and innovative change of the system of cement production should take place.

**Figure 2:**
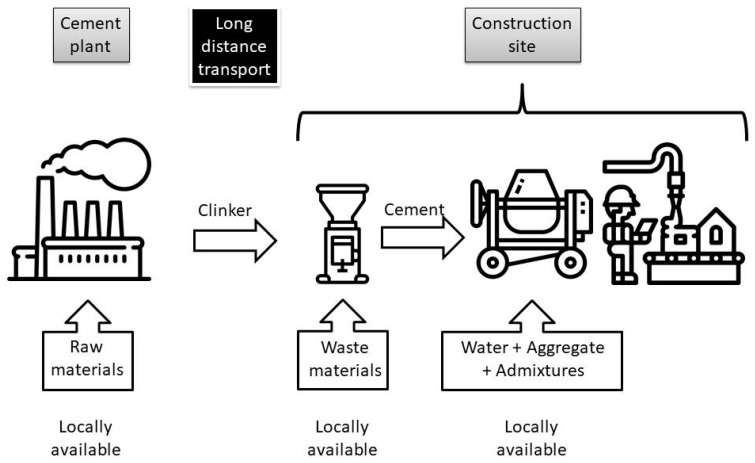
Postulated cycle of materials circulation during cement and concrete production (see Acknowledgments).

The new production system should significantly lower required amount of long-distance transportation and enable usage of all possible local waste materials independently of the available volume. In authors’ opinion cement industry should adopt partially centralized production system which is schematically presented in Figure 2. Cement plants should be focused on producing clinker from local raw materials as they did 70 years ago. Clinker would be transported for a long-distance to a construction site (or ready-mix producer). At a construction site a simple grinding device (e.g., basic industrial ball mill) would be located. Only waste materials located in an immediate neighborhood to a construction site would be sourced and used for blended cement in situ production. The production process would be based on grinding clinker with waste materials in proportions depending on quality, volume and type of ground waste materials mix. The grinding procedure would also influence the strength characteristics of achieved blended cement through the size of a diameter of a cement grain.

The in situ production of blended cement would take place alongside demand. All produced cement would be used instantly for the creation of a fresh concrete mix. No cement would be warehoused at any time preventing degradation of its properties over time. The proposed partially centralized production system is characterized by significant green advantages in comparison to the ordinary centralized production system, but it is also associated with some technological challenges. The first key advantage of the proposed cement production system is the limitation of long-distance transport of huge volumes of waste materials. In the current system waste materials are transported to cement plant and then as a part of freshly produced cement to a construction site. In the proposed production system, long-distance transportation of waste materials would not exist. Moreover, only clinker (less than 60% of cement mass) would be shipped from a cement plant to a building site. The second key advantage of the proposed cement production system is enabling utilization of all possible waste materials which are locally accessible. Majority of these (limited in volume and scattered in terms of location) waste materials are not used by the centralized cement industry. The third advantage of the proposed production system is possible full customization of the produced blended cement. One can produce cements characterized by very specific properties which are needed on a particular construction site. The last advantage of the proposed production system is that the amount of produced cement would match the exact needs of a construction-site. No cement would be wasted during transportation and storage. Technological challenges would be associated with the consistency of the process of the in situ production. One can imagine a dedicated milling device supported by a computer with a large data base of waste materials and their possible mixes to be used for blended cement production. Testing of an in situ produced cements would be very important too. Nevertheless, bringing the last process of cement production to a building site would create a whole new reality of highly efficient waste materials utilization and full customization of achieved cement.

## 3. Research Program

**Figure 3:**
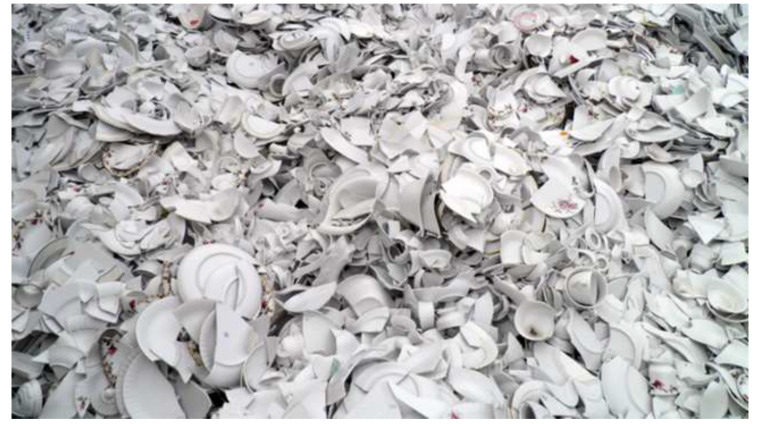
Crushed ceramic pots (waste white ceramics) [24].

**Figure 4:**
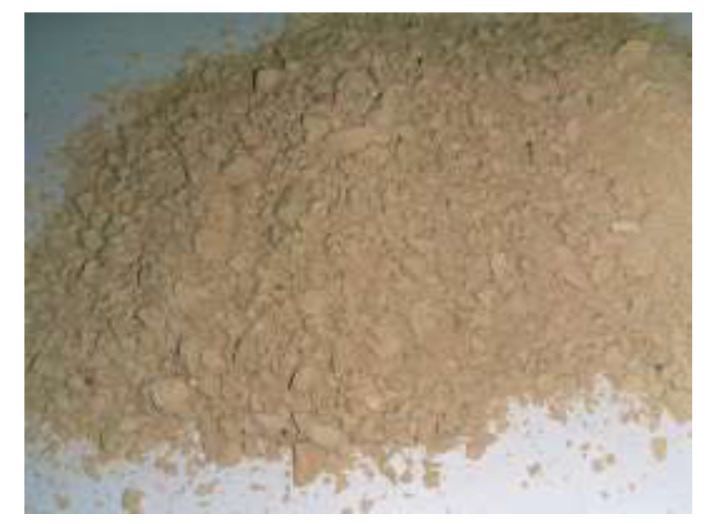
Waste white ceramics mixed with Portland clinker before grinding.

**Figure 5:**
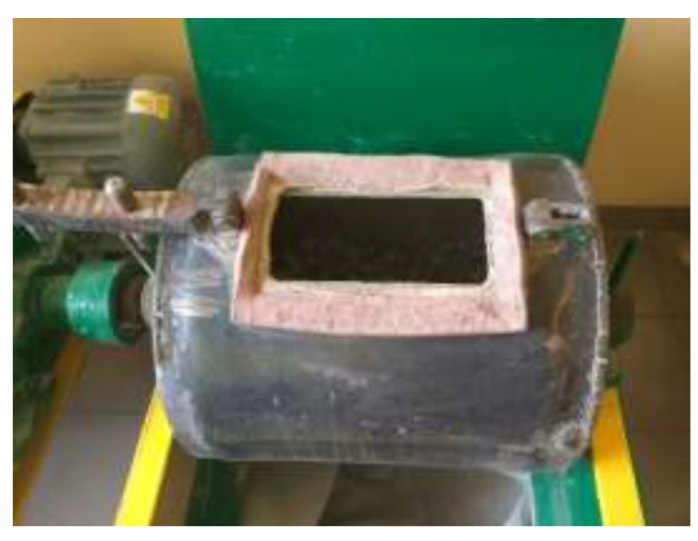
Small ball mill.

The research program was aimed to prove that the concept of in situ production of blended cement according to the proposed partially centralized model is feasible. Raw Portland clinker was sourced from a large cement plant and transported for a long-distance to a laboratory. As a locally available waste material suitable for production of blended cement crushed ceramic pots from a small pottery plant were chosen (Figure 3). One must keep in mind that up to 30% of produced pottery is crushed, damaged or dismissed during production [24]. The scale of waste depends on multiple factors (shape of produced pottery, type of finishing, targeted quality of the end product etc.). White ceramic was chosen as an exemplary waste material. It was selected as the common and well-known waste all over the world [25]. The properties of white ceramic are also similar in different countries in contrary to red ceramics and other popular waste materials (e.g., fly ash, slag etc.). Waste materials available only in certain regions of the world (e.g., rice husk, sea shells, etc.) were not considered for the research program. The waste white ceramic was transported locally to the laboratory. Crushed ceramic pots for blended cement production were mixed with the previously baked Portland clinker (Figure 4). The grinding of the waste white ceramics (WC) and Portland clinker (and small amount of anhydrite as a cement setting time regulator) was carried out using a basic ball mill (Figure 5). Such type of mill is quite common across different industries. The waste white ceramics was dosed in the amounts of 0%, 6% and 12% of clinker mass (code names of cement series C, C6 and C12, respectively). The cements in question were prepared using ball mill loaded with a 5-kg charge. A single charge (by weight) consisted of 95% of clinker and waste white ceramics, 5% of anhydrite. All cement series were obtained by grinding for 45 min. The adopted grinding process was based on authors’ previous experience [24,26]. The detailed ingredient compositions are listed in Table 1. Prepared cements were tested in the raw state and in a form of fresh and hardened standard mortars characterized by w/c = 0.5 and aggregate/cement ratio of 3.0. Standardized sand and tap water (see Table 2) meeting the requirements of EN 1008:2002 were utilized to prepare the mortars. The mortars were mixed and cast just after in situ produced blended cements were ready-to-use. The research program consisted of three phases.

Phase one was dedicated to test achieved blended cements. Morphology and chemical composition of cement using a scanning microscope (SEM, LEO Electron Microscopy Ltd, UK, model 1430 VP) and the fineness of cement by means of Blaine air permeability method according to PN-EN196-6 [27] were tested during this phase. Phase two was focused on properties of fresh mortar. The setting time according to PN-EN196-3 [28] was tested during this phase. The third phase was dedicated to properties of hardened standard mortar. Strength characteristics (PN-EN196-1 [29]) were established in time intervals of 2, 7, 14, 28 and 90 days of maturing of mortar. Both, flexural strength and compressive strength were of interest.

## 4. Results

**Figure 6:**
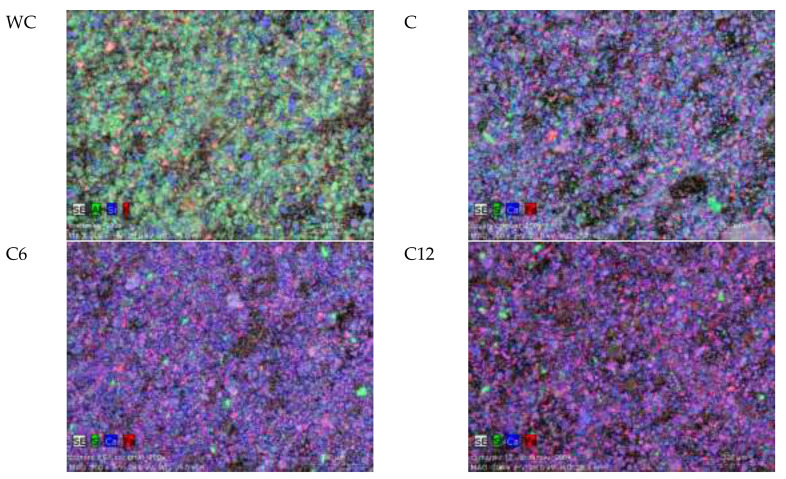
SEM photos (artificial colors) of white ceramic and cements.

**Figure 7:**
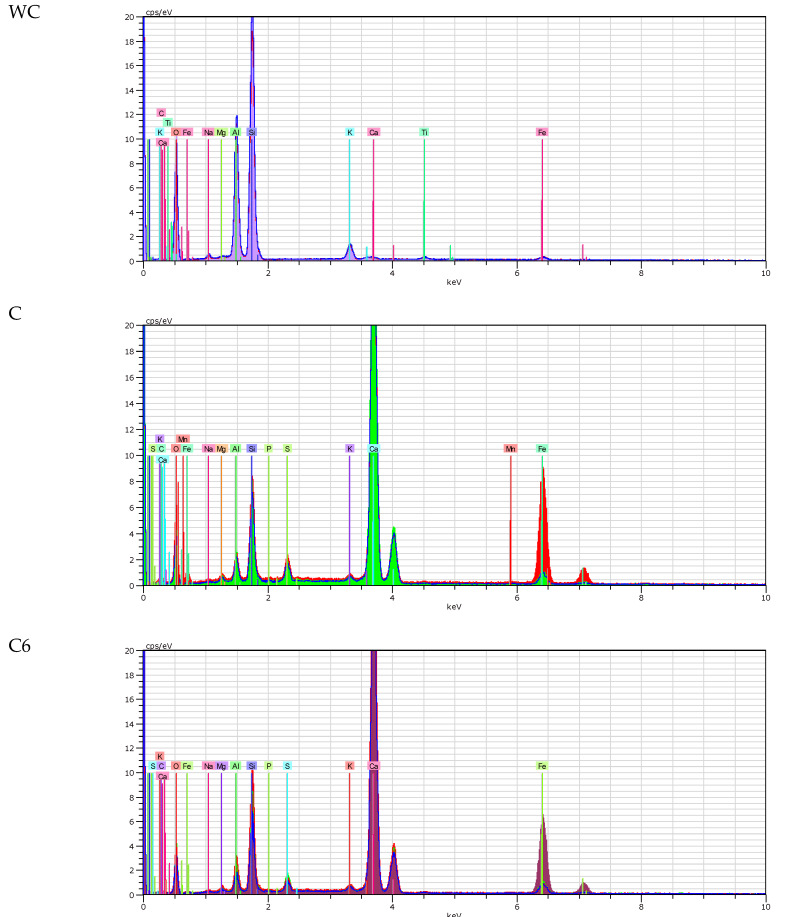

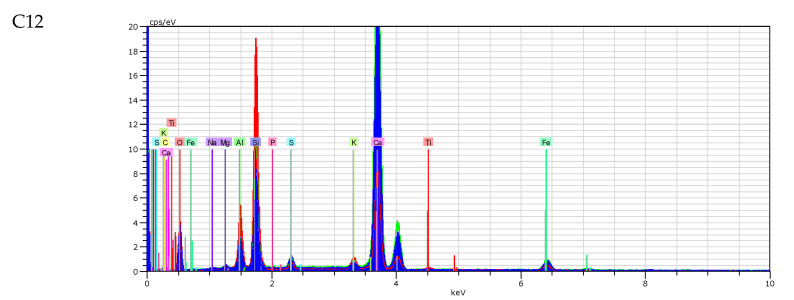
Chemical composition spectra.

SEM analysis was conducted for white ceramics, cement with no white ceramic, blended cement with addition of 6% of ceramics and blended cement with addition of 12% of ceramics. Taken SEM photographs are presented in Figure 6 using artificial colors. The same four samples were used for energy-dispersive X-ray spectroscopy (EDS) analysis. The SEM photos and EDS analyses show that samples with 6% and 12% content of white ceramic differ both from each other as well as in relation to the reference cement sample. These differences are visible both in the composition and microstructure. Based on the results of EDS analyses (see Figure 7), it can be stated that the main elements of tested cements are: calcium, silicon and iron. In addition, the tested cements also contained small amounts of aluminum, sulfur, potassium, carbon, magnesium and phosphorus. Small amounts of titanium were detected in WC and C12 samples. The appearance of titanium in the composition of blended cement confirms the presence of ceramics in the prepared blended cement. Along with the increasing content of ceramics in the cement mix composition, the content of silicon is growing, which is the dominant component of ceramics.

The results of the second phase of the research program are presented in Table 3. The initial setting time was ranging from 95 minutes to 100 min for cement with no white ceramic and with white ceramic, respectively. All three prepared cements were characterized by very similar values of fineness ranging from 5075 to 5120 cm^2^/g. Such small differences should be considered as smaller than measurement accuracy. The results of the examinations of strength characteristics of the cement mortars are presented in Table 4. The addition of white ceramics increased the compressive strength of both blended cements by 8% and 6% for C6 and C12, respectively, after standard 28 days of curing.

The highest compressive strength of 62.5 MPa was achieved by cement C12 after 90 days of curing. This value is by 15.7% higher in comparison to the reference cement, which yielded the compression strength of 54.0 MPa. The control cement was classified as strength class 42.5 R, while blended cements obtained from combined grinding of Portland clinker and waste white ceramic (C6 and C12) can be classified as strength class 52.5 N. Flexural strength was not influenced in a noticeable way.

## 5. Discussion

Cements obtained from combined grinding of Portland clinker—with the addition of waste white ceramic—yielded very satisfactory results for compression strength in each period of testing. One should also remember that the inclusion of clay-based waste improves durability of blended cement [30]. Properties of achieved cement could be further shaped by optimization of a grinding process [31]. The achieved cement proved that the proposed partially centralized system of in situ production of cement is technologically viable. The potential advantages associated with implementing a partially centralized system of cement production both for the construction industry and environment are huge. The volume of materials needed to be transported for long-distances would be significantly reduced. Multiple locally available waste materials would be possible to utilize for cement production. The in situ produced cement would be used immediately after production with no additional transportation required, no storing waste and no waste associated with cement aging. The properties of the produced cement could be influenced in various ways, achieving the binder with characteristics specifically shaped for the local construction site. Preparation of cements characterized by lower strengths than those described by EN 196 (and by other adequate national and international standards) would be feasible. In the same way other key properties of cements (e.g., early strength, heat emission during setting, susceptibility to bleeding, etc.) would be possible to shape on the spot. Some cement production practices which are not feasible in large scale facilities could be successfully adopted. Lack of cement transportation and storing would enable using very fine powders and fumes which are risky to use for traditional cement production which is often associated with long period of time before cement is used for the creation of a specific concrete mix. The proposed approach for in situ cement production will require a new strategy of quality control of cement. The new quality assurance system would be decentralized, but strength margins of produced cement could be much smaller due to its instant usage. This phenomenon would create another way of limiting energy and raw materials consumption during the production of cement. Keeping all the above facts in mind one can see that the proposed in situ system of cement production would significantly influence the CO_2_ emissions associated with its production and circulation in the construction industry. Traditional ways of making cement production greener reached their technological limits. Hence, far, environmental innovations applied to production of cement had a character of sustaining innovation. It is time to get to the next level of innovation in the production of cement and try truly disruptive ideas like the proposed partially centralized system of in situ production of cement.

In the presented production of blended cement only up to 12% of weight was replaced by white ceramics. In Europe (according to EN 197-1) multiple types of blended cements are produced in a traditional fully centralized way (CEM II, III, IV and V). These cements contain from 6% (CEM II/A) to 95% (CEM III/C) of different waste materials. Authors used only white ceramics in reasonably small amounts to prove the concept of feasibility of local cement production. Real life applications of in situ blended cement production would be based on much higher amount of waste than these presented in the study.

The share of the construction market which would be able to harness the proposed cement production system is currently difficult to assess. Available data about waste materials varies from country to country. There are different rules regarding classification of waste materials and ways of dealing with them. One should also remember about rough storing of waste materials and a whole waste ‘black market’ existing in some parts of the world. Based on authors’ experience with using waste materials (waste red ceramic [21], waste white ceramic [26], waste sand [23], raw silica fume [32], waste glass [19], waste steel fiber [22], etc.) for cement and concrete production in Central Europe, at least 30% of building sites and ready-mix producers could instantly implement the proposed system. On the other hand, if the system were implemented, the available resources of waste materials would emerge through using waste which was utilized in other (less efficient) ways. It would use waste which was prone to rough “recycling” practices. The final cement market penetration by the proposed system could easily reach much higher share.

As a truly disruptive technology [33,34], the proposed system is going against existing rules and regulations of the cement and concrete industry. One should also remember that the rules and regulations vary significantly between particular countries. In authors’ opinion the importance of reducing CO_2_ emissions by cement industry will trigger changes (both technological and legal) in cement and concrete production. Organizing a part of the cement production process on-site will open new avenues of technological development. It will also cause significant reduction of CO_2_ emissions and utilization of broad assortment locally available waste materials. As all disruptive technologies the proposed method of blended cement production will shape new technological reality which will force changes in existing rules and regulations.

## 6. Conclusions

The following conclusions can be drawn from the conceptual analysis and research program supporting it:

- Waste white ceramics can be successfully used as a full-value additive in the in situ production of a blended cement;
- The innovative method of combined grinding of Portland clinker and crushed ceramic pots resulted in obtaining cements with considerably higher compressive strength in all tested time intervals;
- The cements obtained from combined milling of clinker and pots were also characterized by a high increase in strength after 28 days. Compressive strength of cement with 6% addition of ceramic pots, tested after 90 days, was higher by 16.3% when compared to the strength determined after 28 days. Similarly, for the cement with 12% addition of pots, the increase was 19.8%, and for reference cement the increase was 12%;
- The research demonstrated that damaged ceramic pots can be used in the cement industry, leading to high economic and environmental benefits in terms of sustainable development in the construction sector;
- The proposed partially centralized model of in situ production of cement should be tested using numerous waste materials and various milling techniques.

## Figures and Tables

**Table 1:** Composition of ball mill charges.

Cement Series	Mass Proportions (kg)		
	Waste White Ceramics	Portland Clinker	Anhydrite
C	0.000	4.750	0.250
C6	0.285	4.465	0.250
C12	0.570	4.180	0.250

**Table 2:** Tap water characteristics in city of Częstochowa.

Parameter	Unit	Value *
Turbidity	NTU	0.38
Color	m/lg/L Pt	<5
pH	–	7.7
Ammonium ion	m/lg/L	<0.05
Nitrite	m/lg/L	<0.018
Nitrates	m/lg/L	34.3
The permanganate index	m/lg/L	<0.50
Chloride	m/lg/L	32.5
Iron	μ/lg/L	46
Manganese	μ/lg/L	<10
Sulfur	m/lg/L	51.7
General hardness	m/lg/L CaCO_3_	212
Basicity	mval/L	2.26

* as provided by water treatment plant.

**Table 3:** Initial setting time and fineness of tested cements.

Cement Series	Initial Setting Time (min)	Fineness (cm^2^/g)
C	95	5090
C6	100	5075
C12	100	5120

**Table 4:** Strength characteristics of tested mortars.

Cement Series	Compressive Strength Determined After Days (MPa)				
	2	7	14	28	90
C	21.3	35.0	40.4	47.5	54.0
C6	23.2	36.6	44.1	51.3	61.3
C12	23.5	35.0	42.2	50.4	62.5
**Cement Series**	**Flexural Strength Determined After Days (MPa)**				
	**2**	**7**	**14**	**28**	**90**
C	4.6	6.3	6.4	6.9	7.7
C6	4.3	6.2	6.6	6.9	7.8
C12	4.2	6.0	6.5	7.9	7.9
